# Social Integration and Residence Intention of Foreigners in Western China: Evidence from Xi’an

**DOI:** 10.3390/ijerph191710519

**Published:** 2022-08-24

**Authors:** Xiaoxian Guo, Qian Zhang, Yaxin Cheng, Jing’en Song

**Affiliations:** 1 Department of Sociology, Institute of Empirical Social Science Research, Xi’an Jiaotong University, Xi’an 710049, China; 2 Department of Sociology, The Chinese University of Hong Kong, Hong Kong, China

**Keywords:** social integration, residence intention, the belt and road initiative, China, Xi’an

## Abstract

Since “Belt and Road Initiative” (BRI) of 2014, the number of foreigners in China has increased rapidly and China has become an importing country for immigrants, a change ongoing since the beginning of the 21st century. To respond to the rapidly increasing number of foreigners in China, the government frequently revised the immigration policies and issued new regulations for foreigners. However, scholars understand very little about how the foreigners perceive their integration into Chinese society or decide to pursue long-term residency or lawful permanent resident status. While some pioneering studies touch on this, with samples from the coastal megacities, no empirical evidence has been collected from smaller, inner cities. Three new findings about the foreigners in Xi’an, a major city in western China, fill this literature gap. First, the level of subjective social integration is largely influenced by the local networks. Second, the level of objective social integration depends largely on local and hometown networks. Third, the intention to obtain long-term and permanent residency in China is more evident in those foreigners who come from countries covered by the BRI and who consider China to be a better place to live than their home country.

## 1. Introduction

Why do foreigners come? How do they live among us? What factors persuade them to stay longer? Our intensive literature search finds that the theoretical and empirical answers to these questions came from observations of North American, European, and Oceanic countries, which led to macrolevel economic explanations, mesolevel insights into the family’s decision to migrate, and microlevel investigations of the migrants’ personal merits and social networks. In the view of contemporary Western scholars, China enters this overall pattern of international migration as a major source of emigrants, which was an accurate understanding of China from the late 19th century to the beginning of the 21st century. As a result, most of the social science researchers perceived those three areas of analysis in international migration to be unrelated to China as a target destination, and there is a clear lack of theoretical insight or data collection on the foreigners in China thus far.

The sixth national census of 2010 revealed 590,000 foreigners living in mainland China (National Bureau of Statistics of China. The Sixth National Population Census in 2010 [EB/OL]. http://www.stats.gov.cn/tjsj/pcsj/rkpc/6rp/indexch.htm, accessed on 1 June 2022).The “Belt and Road Initiative” (BRI) of 2014 created more opportunities, unprecedented convenience, and stronger incentives for international students and employees to come to mainland China. The latest statistics demonstrate a totality of 500,000 international students and 900,000 foreign employees in mainland China [1]. In the seventh national census of 2020, there were nearly 846,000 foreigners living in mainland China (National Bureau of Statistics of China; the Seventh National Population Census in 2020 http://www.stats.gov.cn/tjsj/pcsj/rkpc/7rp/indexch.htm, accessed on 1 June 2022). This means that the number of foreigners increased by almost 50% in the last decade. The number of countries covered by BRI has been growing, and by the end of July 2022, China had signed more than 200 cooperation documents with 149 countries and 32 international organizations (Belt and Road Portal; https://www.yidaiyilu.gov.cn/xwzx/gnxw/269051.htm, accessed on 1 August 2022).

Clearly, China has emerged as an immigrant-receiving country [2]. At the national level, to handle the fast-increasing number of foreign visitors and immigrants to mainland China (to simplify our discussions, “China” in this study always refers to mainland China. The international migration and migration laws of Hong Kong SAR, Macau SAR, and Taiwan Province of the People’s Republic of China are not addressed in this study), the 13th National People’s Congress of the People’s Republic of China passed a bill to establish the National Immigration Administration (NIA) in 2018. In academia, the emergence of this new trend encourages us to address the following two questions, ones that every Western immigration society has been facing for years: (1) what kinds of foreigners in China tend to integrate themselves better into Chinese society? and (2) among the temporary visa-holders, who is more likely to adjust the temporary immigration status to permanent residency? Some of the pioneering studies have provided preliminary answers to these questions by using empirical data from China’s coastal megacities [3,4,5,6]. However, little attention was given thus far to the cities of China’s western inner land. This pioneering research attempts to investigate how the quality of social integration affects the permanent residency intentions of the foreigners living in Xi’an, Shaanxi Province.

Unlike Beijing, Shanghai, or Hong Kong, Xi’an is an unfamiliar name for most of the foreigners. As the provincial capital of Shaanxi, Xi’an is a large city of 12 million residents. Due to its geographical location, Xi’an has been the central hub of economic, political, cultural, and educational resources in China’s western inland since the very beginning of Chinese civilization. Xi’an was the political center of 13 dynasties of ancient China and served as the starting point of the Silk Road since the Han Dynasty. As the home of nine World Heritage Sites, Xi’an defines traditional Chinese culture. Since 2014, Xi’an has started to gain new momentum toward globalization and faster economic development, due to the arrival of the “Belt and Road Initiative” (or the “BRI”). The BRI has brought policy advantages to Xi’an by comprehensively deepening the reforms and opening up movement, such as bringing more international exposure to local history and culture, increasing investments in higher education, and creating more incentives to attract foreign direct investments (FDIs) and international joint ventures. As a result, Xi’an has seen an increasing number of foreigners. In 2017, the number of international students in Xi’an exceeded 10,000. On 4 May 2018, Chen Naixia, spokesperson and deputy secretary of the Higher Education Work Committee of the CPC Shaanxi Provincial Committee, introduced the number of international students in Shaanxi Province at a press conference held as part of a series on the theme of “Strengthening Cultural Construction and Demonstrating Cultural Confidence”, held by the Foreign Propaganda Office of the CPC Shaanxi Provincial Committee (available at http://www.shaanxi.gov.cn/szf/xwfbh/201807/t20180726_1513337_wap.html, accessed on 1 June 2022). Our study focuses closely on this expanding community of international immigrants in Xi’an, a city with a strong emphasis on Chinese tradition.

At the end of 2019, a worldwide outbreak of COVID-19 occurred. International flights were drastically reduced by the impact of COVID-19, and at the height of the epidemic, the international population movements were virtually disrupted. The international flight disruptions, home isolation, and other anti-epidemic policies caused a significant drop in the number of foreign arrivals to China since the end of 2019 and the beginning of 2020. According to the data from China’s National Immigration Administration, 4.531 million foreigners entered and left the country in 2021, down 65.9% from the previous year. COVID-19 leaves us with a very limited study of the foreigners in China. People’s intention to reside permanently and immigrate may also have fluctuated along with this major outbreak. Therefore, the most ideal analytical data for the study of the foreigners in China should be collected before the outbreak.

## 2. Literature Review and Theoretical Analysis

### 2.1. Migrant Studies: Origins and Chinese Studies

The migration research originated in the West, and the classical theories about migration also started in the West [7,8,9], such as push–pull theory, human capital theory, and social network theory [10,11]. Depending on the space for mobility, the migrants can be divided into domestic and international groups. Most of the recent research on domestic migrants in China focuses on the urban and rural migrant populations, and the research results are abundant. The research issues include the identity, social capital, social integration, and residence rights of domestic migrants, with mainstream research focused on migrant workers but also covering different categories of mobile populations, such as small-town youth and college students [12,13,14,15,16,17,18].

In contrast, relatively little attention and research was paid to international migrants living in China. The recent research on international migrant groups in China focused on the factors affecting foreigner integration [6], migration mechanisms [3], migration status [4], and residence patterns [19]. In terms of research objects, more attention was given to the geographical characteristics of the foreigners forming ethnic groups in China, such as the African group in Guangzhou [4], the Korean group in Beijing [20,21], and the Japanese group in the *Gubei* area of Shanghai [22]. By combing through the literature, it is easy to find that the recent research methods on foreigners in China were mostly qualitative studies, and there were relatively few quantitative studies based on survey data, which means that there is still much to explore in the study of international migrants in China through empirical analysis.

### 2.2. Social Integration of Immigrants

Social integration has been the focus of migration research and has gradually developed into a multi-perspective and diversified topic, accumulating a wealth of academic results. Because of the various subjects of social integration and the very complex dynamic process involved, different perspectives were used for different mobile populations, such as internal and international migrants, and related theoretical concepts, such as social assimilation [23,24], social adaptation [8], and social integration [25], are used to explain how immigrants adapt to their new home.

According to the existing research, immigrant integration can be categorized into economic integration [26], social integration [27], political integration [28], and cultural integration [29]. Although scholars have not reached a consensus on the specific dimensions of immigrant integration, a basic consensus is that immigrants’ social integration has multiple dimensions and needs to be examined from multiple perspectives. For example, the “two-dimensional” model represented by Gordon [30] includes structural and cultural dimensions, while the “three-dimensional” model represented by Junger-Tas [24] includes structural, sociocultural, and political–legal dimensions.

These concepts actually represent different theoretical viewpoints. They summarized the adjustment and adaptation of immigrants from different perspectives in the process of integration and investigated the interactive relationship between immigrants and social inflows. These theories are not antagonistic and mutually exclusive, but complementary to each other, so it is difficult to distinguish one from the other when describing the integration process. Social integration itself is a systematic, multisubject, multidimensional, and phased complex process.

A review of the literature shows that the social integration of immigrants is influenced by multiple structural factors. We summarize them into three levels of influence: human capital at the micro level; social capital at the meso level; and the policy system at the macro level, which together have an impact on immigrant integration.

First, human capital theory has a tendency to focus on individualism at the micro level. According to this theory, many of the difficulties encountered by the immigrants in the process of integration are due to their lack of labor skills or clashes with their original, cultural values [31], mainly including education, language skills, work experience, and other factors [8,24]. Second, social capital theory pays attention to the structural factors at the meso level, such as the social network and communication status of immigrants. The immigrants can obtain emotional and instrumental support by building heterogeneous social networks in the places that attract many immigrants on [31]. However, some of the studies have pointed out that selective communication may also prevent migrants from expanding their social networks and enhancing their recognition of the place of entry [32]. Third, policy system theory reflects the structural influence at the macro level. The policies of the countries of immigration will fundamentally affect the degree of social integration of immigrants [33].

### 2.3. Immigrant Intentions for Residency

As one of the divergences proposed by Massey, the originator of migration research, whether the motivation of emigration lies mainly in individuals or structures [34], there are primarily two theoretical explanations to interpret the motivation for migration, namely, the macroscopic perspective and the microscopic perspective. The former principally involves push–pull theory, world system theory, and labor market theory [3]. Push–pull theory is one of the most classic macro theories in migrant research. It emphasizes that negative factors would “push” immigrants to leave their native country or region, and positive factors would “pull” them to live in another nonnative country or region. The immigrants’ decisions are determined by these push–pull factors, which include wage levels, housing conditions, and medical resources. In conclusion, under the combined influence of the push–pull factors, immigrants choose to migrate to maximize their interests [35]. Noticeably, these two factors are not absolutely opposed since there are both pulling and pushing factors in both the inflow and outflow areas, or in other words, both places have attraction and repulsion effects.

In terms of micro theories, the most representative perspectives are human capital theory and social network theory, which emphasize individual features and interactions, respectively. The studies suggest that migration decisions are subject to changing levels of education, skill, and work experience, which is reflected in the fact that immigrants who have received higher education or who are more proficient in the language of their destination have a distinct advantage in accessing and appreciating information [36,37]. Thus, Chiswick [38] proposed the concept of destination-specific human capital, and Liang Yucheng [3] summarized it as the migrant’s ability to obtain knowledge and information from other parts of the immigrant community, stressing that migration-specific human capital plays a different role from human capital in general on decision-making.

The social network theory emphasizes that migrants have built up strong social ties through the various kinship and hometown connections accumulated in the place they emigrate from, and that, after migration, they could gain economic and cultural conveniences through their social networks to facilitate their successful integration, thus increasing their willingness to stay to a certain extent [4,39,40,41].

Much of the existing research on migration and networks emphasizes the role of the local networks formed in immigration, or inflow, destinations and the hometown networks in outflow places on the intention to stay. First, in terms of local networks, it has been found that the deeper and more frequent the interactions are between migrants and the original residents in the inflow destinations, the more obvious migrant willingness is to settle down [11], most notably manifesting as interactions with the Chinese [19]. In terms of hometown networks, two main mechanisms affect migrant intentions. One emphasizes the influence of prior migration experiences of people in the same ethnicity or from the same hometown. As an increasing number of ethnic groups migrate, this effect of hometown networks continues to increase, and the previous experiences among others from the same ethnic group could reduce the cost of migration and adaptation. In other words, the endogeneity of the network gradually drives migration behavior [3]. The other mechanism impacting migrant intentions is the emotional support and risk-averse role of ethnic kin networks [5], as well as the status of interactions with people from the ancestral country in the inflow destination. The companionship of relatives could provide emotional support and play a certain role in shared economic risk, which in turn promotes the intention of migrants to stay. The migrant groups with larger hometown networks and stronger social capital in their ancestral countries have a stronger willingness to stay.

In addition to social networks, both the individual features and social environment factors affect migrant intentions, with the former referring to gender, age, and migration attribute factors and the latter including social structure and living conditions in inflow areas. The existing studies have indicated that men have a stronger willingness to stay [42], that younger groups are more likely to choose to immigrate [43], and that the groups who have stayed longer are more interested in permanent residency [42]. Simultaneously, the foreigners with better Chinese proficiency or from developing countries have a stronger desire for long-term residence [19]. If the migrants have a higher sense of identification with the place from where they immigrated or with its government, their willingness would be stronger [42,44]. Additionally, participation in social activities has an impact on their long-term intentions, as migrants who actively concern themselves with and participate in activities organized by local organizations have a stronger willingness to stay [45]. Not all of the variables have a stable and generally significant effect on the intention to stay, and sometimes inconsistent findings and conclusions will emerge. For instance, some of the studies have found that the women among the transit population are more inclined to settle or immigrate than the men [43]. The significant effect of age and residence time on long-term intention is not a purely linear positive effect but an inverted U-shaped relationship, which results in some scholars exploring the reasons for the differences in the impact of individual features, taking into account the functional nature of the location and the level of social development of different cities [46].

### 2.4. Foreigners in China: Social Integration and Residence Intention

Based on the above literature review, we find that there are still some opacities that require further research. First, most of the existing studies on migrant population groups focus on the urban migrant population, and less attention is given to the foreigners in China. Second, most of the studies are quantitative. Third, most of the research sites are in large coastal cities, such as Beijing, Shanghai, and Guangzhou, and there is almost no research on the foreigners in China in the western and inland areas. In the past, the foreigners in China mainly gathered in large coastal cities. With the promotion of the “Belt and Road Initiative”, an increasing number of foreigners choose to study, work, and do business in inland cities; thus, the number of foreigners in the inland areas is rapidly increasing. The living conditions, integration status, and residence intention of the inland foreigners also deserve attention and are still relatively ignored in empirical studies.

In summary, this study chooses the social integration and residence intention of the foreigners in Xi’an as its primary topic. This paper will explore the social integration status and influence of the foreigners in China from three theoretical levels: macro; meso; and micro. On this basis, it proceeds to measure the foreigners’ long-term residence intentions and migration intentions, choosing the classical push–pull theory as the main perspective to explore the residency of international immigrant groups. The push factors mainly refer to the social structure characteristics of their emigration regions, such as low personal identification with the home country and poor integration in the home country. The pull factors refer to the local social networks and ethnic social networks formed by immigrants in the place of immigration, such as the degree of social integration and identification. The key explanatory variables in this study are social networks, consisting of local and hometown networks, and in analyzing these factors, we also focus on the influence of other independent variables that may affect willingness.

## 3. Data and Variables

### 3.1. Data

This research is based on the Survey on Foreign Residents in China (SFRC) from 2018, which was conducted for a month in the Xi’an Public Security Bureau Exit and Entry Administration. It was difficult to use probability sampling due to the specificity of the foreigner population. We collected questionnaires from every foreigner who came for business to the immigration hall for a month. The survey questionnaire was mainly for the foreigners who came to the hall for immigration formalities. The survey was available in more than fifteen languages, and there were a total of 837 valid responses. The object of this research was the foreigners in Xi’an who are over 18 years old; therefore, 817 valid samples were entered into the empirical model analysis after screening for missing values.

### 3.2. Variables

#### 3.2.1. Dependent Variables

The dependent variables of this research are the social integration and residence intention of the foreigners living in China. For social integration, we employed two indicators, subjective integration and objective integration. The subjective integration was measured using subjective willingness to interact with the Chinese by doing something together. It is measured by classic Bogardus Psychosocial Distance Scale, which specifically includes five items: “Are you willing to do the things listed below: chat with Chinese; work with Chinese; have Chinese neighbors; make friends with Chinese; marry Chinese; or let your children marry Chinese” [47]. The objective integration was measured using actual social participation, such as associations, religious meetings, interest groups, and volunteer meetings. These two variables are continuous variables originally: subjective integration consists of the summation of five Likert-measured variables, that makes it range from 5 to 25; objective integration consists of four dummy variables, that make it range from 0–4. For comparison and interpretation, we divided the subjective and objective integration into three categories: high; middle; and low. The specific descriptions are shown in Table 1.

It is difficult to measure residence intention objectively, since people’s intention is not only subjective but also unsteady or dynamic, that could change from time to time. Besides, it could show some inconsistencies between what people report as their intention to live or immigrate, and what they actually do (such as submit an application maybe years after). Therefore, we used two subjectively variables to analyze residence intention in this study: long-term residence intention and immigration intention. The former is a binary variable, whose values include residence intention and no residence intention. The latter is a multicategory variable, with values about nonimmigration intention, immigration intention before arriving in China, and immigration intention after arriving in China.

#### 3.2.2. Independent Variables

The key independent variable of our study is social networks, which include local networks and hometown networks.

The local network refers to the number of Chinese people who the foreigners know and connect with in China, which is divided into three categories: small (10 people and below); middle (11 to 50 people); and large (51 people and above). The hometown networks, which mean the connection with the friends and relatives from the home country in this study, consist mainly of the scale of the hometown networks, relatives’ experience, and relative companionship. Similarly, the size of the hometown networks is also divided into three categories, namely, small refers to a network of five or fewer people from their home country in China; middle refers to one of 6 to 50 people; and large refers to one of 51 or more people in China. Relatives’ experience refers to whether the relatives of foreigners have ever lived or worked in China. The companionship of relatives refers to whether the foreigner’s partner, children, parents, or relatives are currently living in China together.

Additionally, this study focuses on the impact of three other dimensions of variables on the intention to stay: inclusive individual characteristics; social background in places of emigration; and living conditions in places of immigration. Individual characteristics include human capital variables and migration attribute variables. The social background in the emigration place mainly includes both home country variables and hometown attribute variables. The living conditions in the immigration place consist of integration variables and recognition variables, and the control variables of this study refer to gender and age. The descriptive statistical results of all of the variables are shown in Table 1.

Table 1 shows that the foreigners living in Xi’an have some group characteristics; for example, most of them are male and young. The proportion of students among the immigrants is as high as over 80%. In terms of migration human capital, most of the foreigners know a little Chinese, but the overall level is relatively poor. For migration, these foreigners have lived in China for about fourteen months on average, but nearly 40% have stayed for only 3 months or fewer, most of whom are international students who have just arrived in Xi’an and started studying abroad. Regarding the social background in the emigration place, there are more foreigners from developing countries and Asian countries, and at the same time, most of the foreigners’ hometowns are in urban areas with a high individual status identity. Regarding the living conditions in the immigration place, the foreigners have a high degree of recognition of China and a high degree of social integration. Ultimately, regarding the key variables that we are concerned about, only a small number of the foreigners’ relatives have lived in China or are currently accompanying foreigners in China. Overall, the foreigners are likely to know more people from their home country than from China; for instance, nearly 80% of the foreigners are at a middle level or above in terms of the size of their hometown networks, and approximately half of the foreigners are at a relatively small level in local networks.

## 4. Results

### 4.1. Subjective Social Integration Model

This study analyzes the influencing factors of international migrants’ subjective integration through the nested model (Table 2). Among them, age, the geographic region of the home country, Chinese proficiency, the time since arrival in China, and the size of the network with Chinese or native people all have a significant influence on their willingness to communicate. With increasing age, the international immigrants’ willingness to communicate increases. The international immigrants from Asia have a low willingness to communicate, which may be due to the close geographical proximity, similar culture, and close ties between Asian countries and China, so the international immigrants from this region are more familiar with Chinese people. The international immigrants who are fluent in Chinese have a higher level of willingness to communicate, can master the local language, and better achieve cultural adaptation. The international immigrants who have been in China for a long time have a lower willingness to communicate, and they may face more cultural impacts and conflicts. The international immigrants with a large network of Chinese people have a higher willingness to communicate, because making friends with local people can help them obtain more heterogeneous information. In contrast, international immigrants who have a large network with people from their own country are less willing to communicate with locals, and the limited size of the social network with native people restricts them from expanding the scope of their communication in China and hinders their social integration process.

### 4.2. Objective Social Integration Model

At the same time, this study analyzes the influencing factors of international immigrants’ objective integration (activity participation) through a nested model (Table 3). Similarly, gender, age, development level of the home country, Chinese proficiency, frequency of coming to China, and network scale with Chinese or native people all have a significant influence on their participation in activities. Men are more willing and more active in participation in various social activities. With increasing age, the participation level of the international immigrants decreases. The international immigrants from developed countries have a lower level of participation in activities, which may be because the level of social development in the developing countries is more similar to that of China, so international immigrants from developing countries are more adapted to China’s social environment. The higher the Chinese language proficiency of the international immigrants is, the higher their level of participation in activities, and their language advantages can help them participate in activities more conveniently. With an increase in the number of visits to China, the participation level of international immigrants also improves. The participation level of international migrants with large networks with Chinese or native people is high, and communication with locals or native people can help them obtain more information and social support.

### 4.3. Residence Intention of Foreigners in Xi’an

As shown in Table 4, there is a significant difference in the distribution between the long-term residence willingness and permanent residence willingness of the foreigners in Xi’an. In general, the level of intention to reside long-term in China is higher than the level of intention to stay permanently; that is, approximately 60% of the foreigners have the willingness to stay in China for a long time (63.56%), and approximately 30% of foreigners have considered applying to the Chinese government for permanent residency (32.84%).

The second part of this research is based on an empirical analysis of the foreigners living in Xi’an; therefore, in the current context of the BRI, we have further analyzed the different influencing factors on the intention to stay between BRI countries and non-BRI countries. Notably, the samples of the BRI countries are based on the list of countries that have signed cooperation documents with China to jointly build the BRI, which was published on the official website of the Belt and Road Portal. From our data, 84% of the foreigners are from the countries along the Belt and Road, and 16% of the foreigners are from non-BRI countries. The analysis started by measuring long-term residence willingness and permanent residence willingness, and when processing, the permanent residence intention variable is turned into a binary variable, which means that its values include naturalization intention and not. It can be found that the foreigners from the BRI countries have higher long-term residence desires than the foreigners from the non-BRI countries. In particular, 55.08% of the foreigners from the non-BRI countries expressed their intention to stay in China for a long time, while 65.17% of the foreigners from the BRI countries had a long-term intention to stay in China. In terms of permanent residency intentions, the foreigners from the BRI countries show slightly higher levels than the foreigners from other countries. Specifically, 33.92% of the BRI foreigners have considered permanently immigrating to China, and 27.27% of the foreigners from non-BRI countries have the same intention.

### 4.4. Long-Term Residence Intention Model

Table 5 illustrates the regression model of factors influencing the foreigners’ desire to reside long-term in Xi’an. As revealed in the table, social networks have a significant effect on the foreigners’ intention to stay, after controlling for other variables. This is reflected in the observation that the foreigners with medium-sized local networks have a relatively strong intention to stay long-term; the foreigners whose relatives have come to China also have a stronger intention to reside on a long-term basis. It is evident that the foreigners’ choice for long-term residence is the combined effect of local and hometown networks.

The previous migration experience of others in the hometown networks partly contributes to the facilitation of the foreigners’ migration and residence and reduces the cost of migration and integration, while local social interaction also promotes better integration into life in inflow places, thus leading to a higher willingness to stay in China for a long time.

In addition, both the social background of the foreigner’s hometown and the condition of the inflow place have significant effects on their long-term residence intentions, which proves the role of the push and pull theory in international migrants’ residence decision-making. In particular, the foreigners from rural areas have a stronger intention to move permanently, and those with lower individual status identity have a stronger intention to stay for a long time. Xi’an, as a new first-tier city in China, attracts international migrants to China as its living and growing conditions improve. Regarding the place of immigration, the foreigners with a higher recognition of China also have a stronger tendency to stay in China in the long term. Furthermore, this paper finds that the younger foreigners are more likely to stay in China on a long-term basis.

### 4.5. Immigration Intention Model

Table 6 reveals the regression model of the factors influencing the intentions of Xi’an’s foreigners to immigrate permanently, with the sample who never had any immigration intention as the reference group.

The social networks have a significant impact on the foreigners’ permanent residency intentions in China, with a focus on the role of hometown networks. Specifically, the foreigners whose relatives are currently together with them in China have a stronger intention to immigrate permanently before they come to China, and the presence of an intimate hometown network provides both emotional and economic support to migrants, which guarantees the stability of their lives. The foreigners with larger ethnic social networks locally are more willing to immigrate permanently after arriving in China (1.19, *p* < 0.01), suggesting that life around people of the same ethnicity provides social support and convenience to migrants. Conversely, there is no clear evidence in the current data indicating that interaction with the Chinese locals would influence their intention to naturalize.

In addition, the migration of human capital, individual status identity, and recognition of Chinese people have significant effects on the foreigners’ willingness to immigrate permanently to China. Those foreigners who received an education in China after arrival are more likely to immigrate permanently to China, indicating that studying and living in China has enhanced the foreigners’ recognition of Chinese culture, which in turn leads to a stronger willingness to immigrate permanently. The foreigners who have basic Chinese language skills before coming to China also have a relatively strong desire to immigrate permanently.

## 5. Conclusions

Based on a recent survey of the foreigners in Xi’an, this study explores the influences on the social integration and residence intentions of the foreigners in Western China. The key conclusions of the study are summarized as follows.

First, the foreigners in western China, represented as Xi’an, do have some group characteristics. The group is relatively young, predominantly male, and generally equipped with some higher education, and the majority of them are international students who received their education there. Meanwhile, due to the BRI, a large percentage of the foreigners came from developing countries and Asian regions, and most of the foreigners have a high level of confidence about China’s future development and a high level of recognition of China’s development opportunities and security conditions.

Second, the subjective integration intentions of the foreigners in Xi’an is high, while the objective integration level is relatively low. Most of them have come to China for only a short time, or relatively few times, and have smaller networks with Chinese people, which may limit their social integration. Both the quality of the subjective social integration and the objective social integration are influenced by their individual characteristics, structural characteristics, and especially their social networks. The local networks that connect with Chinese people have significant effects on the two regression models, and the hometown networks that connect the foreigners to their native friends influence their objective social integration.

Third, the long-term residence intention of the foreigners is relatively high, but their intention to reside permanently is relatively low. While 60% of the foreigners preferred to continue to live in China in the future, only approximately 30% of the foreigners considered the intention to move permanently, which is consistent with recent research on the willingness of domestic migrants to settle permanently [43]. In the in-depth interviews, some of the foreigners indicated that it is too difficult to obtain permanent residency in China and that the inherently high standard would discourage them from immigrating permanently. There are still prospects for further development in terms of immigration costs, conditions for international migrants to move in, and the attractiveness of the current immigration environment.

## 6. Discussion

The social networks, as a source of traction in the inflow regions, are an important factor influencing the willingness of the foreigners to stay in China. Companionship from one’s hometown and previous experience in China have significant effects on the long-term intentions of the foreigners, while relatives and family play an influential role in social support, thus facilitating settlement. At the same time, the two network types have different mechanisms of action on the intention to settle. That is, local social networks and social integration promote the foreigners’ preference for long-term residency, but when considering permanent residency, the social network of one’s own ethnicity is an important factor in promoting their intention to settle.

Last, there are some limitations in this paper. The data used for the empirical analysis are cross-sectional survey data from 2018, and the recent enactment of the Foreigner Permanent Residence Regulations has generated much social debate; these conditions would have enabled a meaningful longitudinal comparative analysis using the latest survey data. Unfortunately, COVID-19 led to a severe restriction of international population mobility, and our foreigner survey project had to be suspended temporarily. We are interested in conducting studies, where possible, on the impact of the dramatic and unexpected event of COVID-19 on the integration, residence intentions, and immigrant intentions of international migrants. Does an outbreak reduce people’s social interactions, and does it also reduce their subjective and objective social integration? Did this outbreak reduce people’s mobility, did it also reduce people’s intention to migrate? In addition, we will combine qualitative and quantitative approaches as much as possible in our future research, utilizing quantitative research based on survey data to explore group patterns, and qualitative research to thoroughly explore the stories behind the representative individuals to effectively provide theoretical and data support for foreigner studies.

## Figures and Tables

**Table 1:** Description Table of Variables (*n* = 817).

Variables	Mean	Standard Deviation	Variable Description
***Dependent variable***			
Subjective integration	-	-	Low (2.75%), Middle (35.29%), High (61.96%)
Objective integration	-	-	Low (75.84%), Middle (14.03%), High (10.13%)
Long-term residence intention	-	-	No (36.44%), Yes (63.56%)
Permanent residence intention	-	-	Never (67.16%), Considered before coming China (15.22%) Considered during the stay in China (17.62%)
***Independent variable***			
*Social networks*			
Local networks			
Network Size	-	-	Small (50.52%), Middle (31.50%), Large (17.98%)
Hometown networks			
Network Size	-	-	Small (24.38%), Middle (51.38%), Large (24.25%)
Relative companionship	0.27	0.44	Yes = 1, No = 0
Relative experience	0.24	0.43	Yes = 1, No = 0
*Individual characteristics*			
Gender	0.71	0.45	Male = 1, Female = 0
Age	24.35	6.40	[18, 68]
Migration Human Capital			
Access to Chinese education	0.69	0.46	Yes = 1, No = 0
Chinese proficiency	-	-	No ability (15.77%), Some skill (45.34%), Good (31.27%), Fluent (7.62%)
Migration Attribute			
Length of stay in China (Month)	14.25	15.76	[1, 60]
*Social background in emigration place*			
Background of home country			
Development level	0.11	0.31	Developed = 1, Developing = 0
Region	0.64	0.48	Asia = 1, Non-Asia = 0
Hometown attributes			
Urban and rural attributes	0.91	0.29	Urban = 1, Rural = 0
Individual status identity	-	-	High (41.38%), Middle (55.45%), Low (3.17%)
*Living conditions in immigration place*			
Subjective integration	19.30	3.78	[5, 25]
Objective integration	0.85	1.07	[0, 4]
Comparison of China and native countries	10.70	2.09	[5, 15]

**Table 2:** Subjective Social Integration Model. (O-Logit Regression Model).

Variable	Individual Feature Model		Structural Feature Model		Integration Ability Model	
	Coef.	Std Err	Coef.	Std Err	Coef.	Std Err
**Individual characteristics**						
Gender (Male = 1)	0.07	(0.17)	0.09	(0.18)	0.04	(0.19)
Age	0.05 **	(0.02)	0.05 *	(0.02)	0.05 *	(0.02)
Marriage	−0.10	(0.23)	−0.20	(0.24)	−0.29	(0.26)
Health (Reference group: Fair)						
Healthy	−0.28	(0.30)	−0.34	(0.30)	−0.40	(0.31)
Very healthy	0.16	(0.30)	−0.00	(0.31)	−0.10	(0.32)
**Structural Characteristics**						
Development level of home country (Developed country = 1)			0.17	(0.30)	−0.05	(0.31)
Region of home country (Asia = 1)			−0.55 **	(0.18)	−0.57 **	(0.19)
Social attribute in hometown (Urban = 1)			−0.29	(0.28)	−0.25	(0.29)
Individual status identity (Reference Group: Low)						
Middle			−0.10	(0.46)	−0.23	(0.47)
High			0.07	(0.47)	−0.04	(0.48)
Integration ability						
Chinese proficiency (Reference group: No ability)						
Some skill					0.32	(0.25)
Good					0.37	(0.28)
Fluent					1.26 **	(0.43)
Times of visits to China (Reference group: once)						
2-5 times					−0.14	(0.20)
6 times and above					−0.11	(0.35)
Length of stay in China (Month) **Social Networks**					−0.02 **	(0.01)
Network size with Chinese people (Reference group: Small)						
Middle					0.31 ^!^	(0.19)
Large					0.93 **	(0.26)
Network size with native people (Reference group: Small)						
Middle					−0.30	(0.21)
Large					−0.55 *	(0.26)
Relatives’ companionship (Yes = 1)					0.12	(0.20)
cut1	−2.46 **	(0.52)	−3.24 **	(0.74)	−3.56 **	(0.83)
cut2	0.62	(0.47)	−0.14	(0.71)	−0.27	(0.80)
*n*	716		713		710	
Pseudo R^2^	0.02		0.03		0.06	

^!^ *p* < 0.1, * *p* < 0.05, ** *p* < 0.01.

**Table 3:** Objective Social Integration Model. (O-Logit Regression Model).

Variable	Individual Feature Model		Structural Feature Model		Integration Ability Model	
	Coef.	Std Err	Coef.	Std Err	Coef.	Std Err
**Individual characteristics**						
Gender (Male = 1)	0.57 **	(0.21)	0.50 *	(0.21)	0.40 ^!^	(0.23)
Age	−0.04 ^!^	(0.02)	−0.03	(0.02)	−0.02	(0.02)
Marriage	−0.21	(0.28)	−0.12	(0.28)	−0.25	(0.31)
Health (Reference group: Fair)						
Healthy	−0.16	(0.33)	−0.09	(0.33)	−0.00	(0.36)
Very healthy	−0.21	(0.33)	−0.09	(0.33)	−0.13	(0.36)
**Structural Characteristics**						
Development level of home country (Developed country = 1)			−0.98 *	(0.43)	−0.63	(0.45)
Region of home country (Asia = 1)			0.24	(0.20)	0.29	(0.22)
Social attribute in hometown (Urban = 1)			−0.08	(0.30)	0.04	(0.32)
Individual status identity (Reference Group: Low)						
Middle			−0.07	(0.47)	−0.15	(0.51)
High			−0.19	(0.48)	−0.34	(0.52)
Integration ability						
Chinese proficiency (Reference group: No ability)						
Some skill					0.93 *	(0.41)
Good					1.21 **	(0.43)
Fluent					0.96 ^!^	(0.52)
Times of visits to China (Reference group: once)						
2-5 times					0.45 *	(0.22)
6 times and above					0.27	(0.38)
Length of stay in China (Month)					0.01	(0.01)
**Social Networks**						
Network size with Chinese people (Reference group: Small)						
Middle					0.93 **	(0.22)
Large					0.99 **	(0.27)
Network size with native people (Reference group: Small)						
Middle					0.81 **	(0.31)
Large					1.07 **	(0.34)
Relatives’ companionship (Yes = 1)					0.08	(0.22)
cut1:	0.44	(0.54)	0.48	(0.78)	3.50 **	(0.96)
cut2:	1.53 **	(0.55)	1.59 *	(0.78)	4.72 **	(0.97)
*n*	720		715		713	
Pseudo R^2^	0.01		0.02		0.11	

^!^ *p* < 0.1, * *p* < 0.05, ** *p* < 0.01.

**Table 4:** Distribution of residence intentions of foreigners in Xi’an.

	Total Sample		Sample of BRI Countries		Sample of Non-BRI Countries	
	Frequency	Percentage	Frequency	Percentage	Frequency	Percentage
Long-term residency intention						
Yes	471	63.56%	406	65.17%	65	55.08%
No	270	36.44%	217	34.83%	53	44.92%
Total	741	100%	623	100%	118	100%
Permanent residency intention						
Yes	246	32.84%	213	33.92%	33	27.27%
No	503	67.16%	415	66.08%	88	72.73%
Total	749	100%	628	100%	121	100%

**Table 5:** Long-term Residence Intention Model. (Logit Regression Model).

	Coef.	Std Err
**Individual characteristics**		
Gender (Male = 1)	−0.05	(0.22)
Age	−0.03 ^!^	(0.02)
**Social networks**		
Network size with Chinese people (Reference group: Small)		
Middle	0.57 *	(0.24)
Large	0.25	(0.29)
Network size with native people (Reference group: Small)		
Middle	−0.14	(0.25)
Large	−0.24	(0.31)
Relative’s companionship (Yes = 1)	0.25	(0.24)
Relative’s experience (Yes = 1)	0.44 ^!^	(0.25)
Structural Characteristics		
Access to Chinese education (Yes = 1)	0.37	(0.23)
Chinese proficiency (Reference group: No ability)		
Some skill	0.24	(0.31)
Good	0.24	(0.35)
Fluent	−0.00	(0.48)
Length of stay in China (Month)	−0.01^!^	(0.01)
**Social background in emigration place**		
Development level of home country (Developed country = 1)	−0.50	(0.35)
Region of home country (Asia = 1)	−0.08	(0.22)
Social attribute in hometown (Urban = 1)	−0.62 ^!^	(0.35)
Individual status identity (Reference Group: Low)		
Middle	−0.50	(0.59)
High	−1.03 ^!^	(0.60)
**Living conditions in immigration place**		
Subjective integration	−0.07 *	(0.03)
Objective integration	−0.12	(0.10)
Comparison of China and native countries	0.10 *	(0.05)
cons	3.28 *	(1.32)
*n*	530	
Pseudo R^2^	0.069	

^!^ *p* < 0.1, * *p* < 0.05.

**Table 6:** Immigration Intention Model.(M-Logit Regression Model).

Never Thought about Immigration as Reference Group	Considered before Coming China		Considered during the Stay in China	
	Coef.	Std Err	Coef.	Std Err
**Individual characteristics**				
Gender (Male = 1)	1.14 ***	(0.34)	0.37	(0.29)
Age	−0.03	(0.03)	0.02	(0.02)
**Social networks**				
Network size with Chinese people (Reference group: Small)				
Middle	−0.09	(0.31)	0.06	(0.30)
Large	−0.29	(0.40)	0.08	(0.35)
Network size with native people (Reference group: Small)				
Middle	−0.12	(0.32)	0.36	(0.35)
Large	0.08	(0.42)	1.19 **	(0.40)
Relative’s companionship (Yes = 1)	0.86 **	(0.30)	−0.04	(0.30)
Relative’s experience (Yes = 1)	0.00	(0.33)	0.31	(0.30)
**Structural Characteristics**				
Access to Chinese education (Yes = 1)	−0.32	(0.29)	0.68 *	(0.32)
Chinese proficiency (Reference group: No ability)				
Some skill	0.35	(0.42)	0.16	(0.44)
Good	0.87 ^!^	(0.46)	0.67	(0.47)
Fluent	−0.44	(0.76)	0.22	(0.61)
Length of stay in China (Month)	−0.01	(0.01)	−0.01	(0.01)
**Social background in emigration place**				
Development level of home country (Developed country = 1)	−0.49	(0.56)	0.71	(0.43)
Region of home country (Asia = 1)	0.11	(0.29)	0.06	(0.28)
Social attribute in hometown (Urban = 1)	−0.42	(0.42)	−0.28	(0.39)
Individual status identity (Reference Group: Low)				
Middle	−0.24	(0.63)	0.77	(0.79)
High	0.12	(0.64)	0.39	(0.80)
**Living conditions in immigration place**				
Subjective integration	0.01	(0.04)	0.04	(0.03)
Objective integration	0.11	(0.14)	−0.09	(0.12)
Comparison of China and native countries	0.08	(0.06)	0.08	(0.06)
cons	−3.19 ^!^	(1.76)	−4.78 **	(1.68)
*n*	533			
Pseudo R^2^	0.083			

^!^ *p* < 0.1, * *p* < 0.05, ** *p* < 0.01, *** *p* < 0.001.

## Data Availability

The data presented in this study are available on reasonable request from the corresponding author.
